
JOURNAL INFORMATION
==============================
NLM Title Abbreviation: ScientificWorldJournal
Journal ID: ScientificWorldJournal
NLM Journal ID: 101131163
Journal ID: TSWJ

The Scientific World Journal

ISSN: 2356-6140
EISSN: 1537-744X
Publisher: Wiley

ARTICLE INFORMATION
==============================
PMCID: PMC5917209
PMID: 16583116
DOI: 10.1100/tsw.2006.80
Article ID: 948245
Article version: 1
Subjects: Directions in Science

The Two Thrombospondin Type I Repeat Domains of HB-GAM Display a Cooperative Function in N-syndecan Binding and Regulation of Synaptic Plasticity

Raulo Erkki 1 *
Tumova Sarka 1
Pavlov Ivan 2
Hienola Anni 1
Lauri Sari 1
Taira Tomi 1
Rauvala Heikki 1
1 Neuroscience Center, University of Helsinki, Finland
2 Institute of Neurology, University College London, United Kingdom
3 Laboratory of Molecular Neurobiology Neuroscience Center University of Helsinki, Finland
*Erkki Raulo: erkki.raulo@helsinki.fiAcademic Editor: Martin Goette

Publication date: 2006
Electronic publication date: 2006 Mar 29
Volume: 6
First page: 406
Last page: 409
Received 2006 Mar 15; Accepted 2006 Mar 21
Copyright: Copyright © 2006 Erkki Raulo et al.
Copyright year: 2006
License: This is an open access article distributed under the Creative Commons Attribution License, which permits unrestricted use, distribution, and reproduction in any medium, provided the original work is properly cited.
License URL: https://creativecommons.org/licenses/by/3.0/

Keywords: syndecan, TSR, HB-GAM, brain plasticity

==============================
Directions in Science
*TheScientificWorldJOURNAL* (2006) 6, 406–409
ISSN 1537-744X; DOI 10.1100/tsw.2006.80

TheScientificWorldJOURNAL
www.thescientificworld.com

### The Two Thrombospondin Type I Repeat Domains of HB-GAM Display a Cooperative Function in N-Syndecan Binding and Regulation of Synaptic Plasticity

Erkki Raulo[1,]*, Sarka Tumova[1], Ivan Pavlov[2], Anni Hienola[1], Sari Lauri[1], Tomi Taira[1], and Heikki Rauvala[1]

[1]*Neuroscience Center, University of Helsinki, Finland and* [2]*The Institute of Neurology, University College London, Great Britain*

**E-mail**: erkki.raulo@helsinki.fi; sarka.tumova@helsinki.fi; i.pavlov@ion.ucl.ac.uk; anni.hienola@helsinki.fi; sari.lauri@helsinki.fi; tomi.taira@helsinki.fi; heikki.rauvala@helsinki.fi

*Received March 15, 2006; Accepted March 21, 2006; Published March 29, 2006*

**KEYWORDS**: syndecan, TSR, HB-GAM, brain plasticity

The TSR (thrombospondin type I repeat) is defined by a conserved tryptophan/cysteine motif and found in a large family of extracellular matrix and cell surface proteins (for review, see [1]). These proteins include thrombospondins 1 and 2, F-spondin, mindin, and semaphorins F and G. HB-GAM (heparin-binding growth-associated molecule, also designated as pleiotrophin) and MK (midgestation-kidney protein) form a subfamily within this type of proteins and have very similar structures (see Fig. 1 for essential structural features of HB-GAM and MK). Structures of the TSR domains of the thrombospondins have been recently resolved using X-ray crystallography[2], and secondary structures of MK[3] and HB-GAM[4] are currently known based on NMR spectroscopy. HB-GAM and MK fold in a very similar manner, but their TSR domains apparently display significant differences from the TSR structures resolved for the thrombospondins. In general, TSR proteins are implicated in cell surface/matrix binding and regulate migratory responses in many types of cells (for reviews, see [1,5]). A wide variety of TSR proteins are highly expressed in the nervous system, but their functional roles and possible significance of the TSRs in these proteins remain elusive.

Structure-function studies of HB-GAM[6] have recently shown that the two TSRs of the protein mediate binding to heparin/HS (heparan sulfate). In hippocampal neurons, N-syndecan (neural syndecan, syndecan-3) is the carrier of the HS chains interacting with HB-GAM. Furthermore, studies using hippocampal neurons and slices implicate the di-TSR domain in the regulation of neurite outgrowth/plasticity in the developing and adult hippocampus.

The rationale of the structure-function studies using neurite outgrowth/plasticity as a readout derives from previous studies where HB-GAM was shown to enhance neurite extension in hippocampal neurons when presented as a matrix-bound form[7,8] and to inhibit it when an excess of HB-GAM occurs in the culture medium of cells[8]. Furthermore, HB-GAM was shown to be a neuronal activity-induced gene in the hippocampus[9] which, together with the effects on hippocampal neurons *in vitro*, suggested a role for HB-GAM in the regulation of hippocampal plasticity. To study the possible role of HB-GAM in synaptic plasticity in the hippocampus, its effects on LTP (long-term potentiation; long-lasting, activity-induced

*Corresponding author
©2006 with author.
Published by TheScientificWorld, Ltd.; www.thescientificworld.com

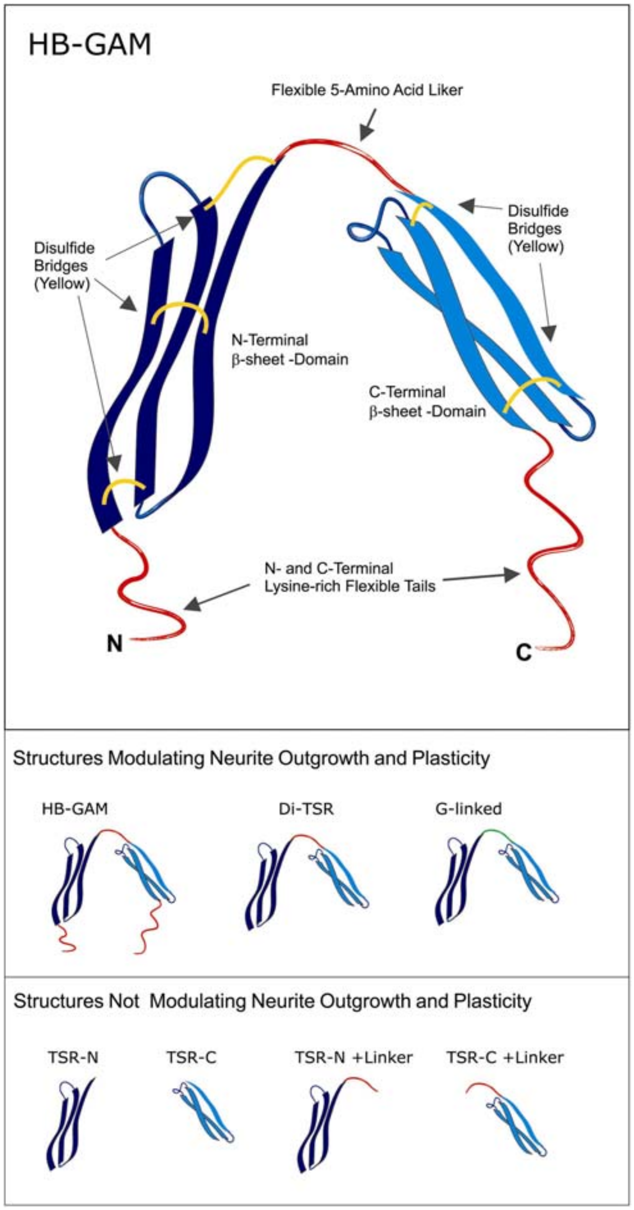

**FIGURE 1**. Schematic presentation of structural features of HB-GAM. Structure/function dissection reveals a cooperative function of the two TSR domains in heparin/HS-binding modulating neurite outgrowth and synaptic plasticity. General characteristics of native HB-GAM molecule are shown (top panel). The biologically informative structure requires the presence of both TSR domains as implicated in the two lower panels.

enhancement of synaptic strength) were studied. Injection of recombinant HB-GAM into hippocampal slices[10] and overexpression of HB-GAM in transgenic mice[11] were shown to inhibit LTP in area CA1. In contrast, HB-GAM knockout mice were shown to display an enhanced LTP[11,12]. In agreement with studies on HB-GAM, N-syndecan was also implicated in the regulation of synaptic plasticity[13]. N-syndecan knockout mice were shown to display an enhanced LTP[11] in a manner shown for its HB-GAM ligand. Furthermore, N-syndecan knockout slices were shown to be insensitive to HB-GAM–induced LTP inhibition[11]. HB-GAM/N-syndecan thus appear to form a unique ligand/receptor pair that is induced by neuronal activity, but inhibits LTP. Since hippocampal LTP is a plasticity paradigm regarded relevant for memory and learning, the HB-GAM and N-syndecan mutant mice were studied for their performance in learning and memory tests. Behavioral testing suggested that the plasticity regulation through HB-GAM/N-syndecan[11,14] is important for learning and memory.

Since HB-GAM regulates hippocampal plasticity through binding to the HS chains of N-syndecan, in our most recent work, we have produced a series of HB-GAM fragments by PCR mutagenesis and studied their binding to heparin/HS and their roles in LTP. Binding studies to heparin/HS (the ectodomain of N-syndecan) were carried out using surface plasmon resonance and ELISA-type assays[6]. The di-TSR fragment had essentially the same affinity to heparin/HS as the intact HB-GAM. Therefore, the polylysine-type N- and C-terminal random coils of HB-GAM are not required for high-affinity binding to heparin/HS. Replacing the naturally occurring flexible linker between the TSR-type domains with glycines did not change the affinity. In contrast to the naturally occurring di-TSR domain or the di-TSR domain having the glycine linker, the single TSR domains alone or with the linker region (Fig. 1) displayed only low-affinity binding; the $K_D$ values of the single domains were several orders of magnitude higher compared to the di-TSR domains or the intact HB-GAM. Therefore, the TSR-type β-sheet areas of HB-GAM cooperate to mediate high-affinity binding to heparin/HS. The linker region between these domains does not directly participate in the binding, but is required to link the TSR-type areas close to each other. The same protein mutants were assayed for their ability to modulate neurite extension in hippocampal neurons and LTP in hippocampal slices. As in the binding studies to heparin/HS, the di-TSR region was shown to be responsible for the effects on neurite extension and for LTP inhibition. These studies agree with the previous findings that N-syndecan acts as an HB-GAM receptor that is required to modulate neuronal connections[8,15] and functional plasticity[11,13], at least when LTP of the Schaffer collateral-CA1 synapses of the hippocampus is used to study activity-induced synaptic plasticity. Structural modulation of neuronal connectivity by HB-GAM/N-syndecan is expected to contribute to functional plasticity, but is an area warranting further experimentation.

Ligation of N-syndecan by HB-GAM regulates cytoskeleton through a signaling pathway that involves cortactin and src-type kinase[5,15]. The N-syndecan–mediated transmembrane signaling in neurons regulates synaptic plasticity that appears to be physiologically important: Disruption of the HB-GAM/N-syndecan pathway compromises performance in learning and memory tests. We therefore regarded it to be of interest to carry out structure-function studies of HB-GAM/N-syndecan interactions. These studies show that HB-GAM binds to the HS chains of N-syndecan and regulates synaptic plasticity through a cooperative action of its two TSR-type domains. Interestingly, the lysine-rich tails of HB-GAM contain multiple matches to the XBBXBX or XBBBXXBX consensus sequences (B is a basic amino acid residue) for heparin/HS binding[16], yet these sequences of HB-GAM are not required for high-affinity binding to heparin/HS. Therefore, heparin/HS binding sites predicted from only sequence data should be taken with great caution. The finding that a cooperative binding of TSR-type domains to heparin/HS regulates synaptic plasticity is of interest from the viewpoint that a multitude of proteins having clusters of TSR-type domains is expressed in the nervous system. Future studies are warranted to answer to the question whether the TSR-type regions of other proteins expressed in the nervous system would display similar features as HB-GAM in their receptor interactions and regulation of synaptic plasticity.

### REFERENCES

1. Adams, J.C. and Tucker, R.P. (2000) The thrombospondin type 1 repeat (TSR) superfamily: diverse proteins with related roles in neuronal development. *Dev. Dyn.* **218**, 280–299.
2. Tan, K., Duquette, M., Liu, J., Dong, Y., Zhang, R., Joachimiak, A., Lawler, J., and Wang, J. (2002) Crystal structure of the TSP-1 type 1 repeats: a novel layered fold and its biological implication. *J. Cell Biol.* **159(2),** 373–382.
3. Iwasaki, W., Nagata, K., Hatanaka, H., Inui, T., Kimura, T., Muramatsu, T., Yoshida, K., Tasumi, M., and Inagaki, F. (1997) Solution structure of midkine, a new heparin-binding growth factor. *EMBO J.* **16**, 6936–6946.
4. Kilpelainen, I., Kaksonen, M., Avikainen, H., Fath, M., Linhardt, R.J., Raulo, E., and Rauvala, H. (2000) Heparin-binding growth-associated molecule contains two heparin-binding beta-sheet domains that are homologous to the thrombospondin type I repeat. *J. Biol. Chem.* **275**, 13564–13570.

5. Rauvala, H., Huttunen, H.J., Fages, C., Kaksonen, M., Kinnunen, T., Imai, S., Raulo, E., and Kilpeläinen, I. (2000) Heparin-binding proteins HB-GAM (pleiotrophin) and amphoterin in the regulation of cell motility. *Matrix Biol.* **19,** 377–387.
6. Raulo, E., Tumova, S., Pavlov, I., Pekkanen, M., Hienola, A., Klankki, E., Kalkkinen, N., Taira, T., Kilpeläinen, I., and Rauvala, H. (2005) The two thrombospondin type I repeat domains of HB-GAM bind to heparin/heparan sulfate and regulate neurite extension and plasticity in hippocampal neurons. *J. Biol. Chem.* **280(50),** 41576–41583.
7. Rauvala, H. (1989) An 18-kd heparin-binding protein of developing brain that is distinct from fibroblast growth factors. *EMBO J.* **8**, 2933–2941.
8. Raulo, E., Chernousov, M.A., Carey, D., Nolo, R., and Rauvala, H. (1993) Isolation of a neuronal cell surface receptor of HB-GAM (heparin-binding growth-associated molecule); identification as N-syndecan (syndecan-3). *J. Biol. Chem.* **269,** 12999–13004.
9. Lauri, S.E., Taira, T., Kaila, K., and Rauvala, H. (1996) Activity-induced enhancement of HB-GAM expression in rat hippocampal slices. *Neuroreport* **7**, 1670–1674.
10. Lauri, S.E., Rauvala, H., Kaila, K., and Taira, T. (1998) Effect of heparin-binding growth-associated molecule (HB-GAM) on synaptic transmission and early LTP in rat hippocampal slices. *Eur. J. Neurosci.* **10,** 188–194.
11. Pavlov, I., Voikar, V., Kaksonen, M., Lauri, S.E., Hienola, A., Taira, T., and Rauvala, H. (2002) Role of heparin-binding growth-associated molecule (HB-GAM) in hippocampal LTP and spatial learning revealed by studies on overexpressing and knockout mice. *Mol. Cell. Neurosci.* **20,** 330–342.
12. Amet, L.E., Lauri, S.E., Hienola, A., Croll, S.D., Lu, Y., Levorse, J.M., Prabhakaran, B., Taira, T., Rauvala, H., and Vogt, T.F. (2001) Enhanced hippocampal long-term potentiation in mice lacking heparin-binding growth-associated molecule. *Mol. Cell Neurosci.* **17**, 1014–1024.
13. Lauri, S.E., Kaukinen, S., Kinnunen, T., Ylinen, A., Imai, S., Kaila, K., Taira, T., and Rauvala, H. (1999) Regulatory role and molecular interactions of a cell-surface heparan sulfate proteoglycan (N-syndecan) in hippocampal long-term potentiation. *J. Neurosci.* **19**, 1226–1235.
14. Kaksonen, M., Pavlov, I., Voikar, V., Lauri, S.E., Hienola, A., Riekki, R., Lakso, M., Taira, T., and Rauvala, H. (2002) Syndecan-3 deficient mice exhibit enhanced LTP and impaired hippocampus-dependent memory. *Mol. Cell. Neurosci.* **21**, 158–172.
15. Kinnunen, T., Kaksonen, M., Saarinen, J., Kalkkinen, N., Peng, H.B., and Rauvala, H. (1998) Cortactin-Src kinase signaling pathway is involved in N-syndecan-dependent neurite outgrowth. *J. Biol. Chem.* **273**, 10702–10708.
16. Cardin, A.D. and Weintraub, H.J.R. (1989) Molecular modeling of protein-glycosaminoglycan interactions. *Arteriosclerosis* **9**, 21–32.

**This article should be cited as follows:**

Raulo E., Tumova, S., Pavlov, I., Hienola, A., Lauri, S., Taira, T., and Rauvala, H. (2006) The two thrombospondin type I repeat domains of HB-GAM display a cooperative function in N-syndecan binding and regulation of synaptic plasticity. *TheScientificWorldJOURNAL* **6**, 406–409. DOI 10.1100/tsw.2006.80.

### BIOSKETCHES

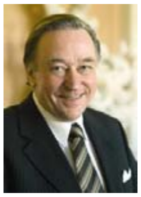

Heikki Rauvala is the director of the Neuroscience Center (see www.helsinki.fi/neurosci) of the University of Helsinki, Finland. He received his MD in 1973 and PhD in 1977 from the University of Helsinki. During his whole career, Heikki Rauvala has been working with cell surface and matrix in cell regulation. The current research topic focuses on the role of extracellular matrix molecules in brain development and plasticity.

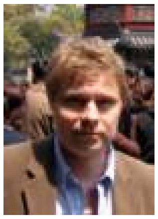

Dr. Erkki Raulo graduated with M.Sc. in biochemistry from the University of Helsinki, Finland in 1994. He received his Ph.D. in molecular neurobiology and biochemistry in 1995 at the University of Helsinki. Currently he continues the research in heparin-binding matrix-proteins of the brain in the Laboratory of Molecular Neurobiology, Neuroscience Center, University of Helsinki. Since 1999, Dr. Raulo has been working as the scientific coordinator of the Helsinki Graduate School in Biotechnology and Molecular Biology administered by the Institute of Biotechnology, University of Helsinki.
